# Navigating the Web of Influence: A Bibliometric Analysis of Social Media Addiction

**DOI:** 10.7759/cureus.62283

**Published:** 2024-06-13

**Authors:** Anish K R, Jesteena Abraham K S, Jobin Jose, Nice Mary Francis P, Ajesh P Joseph

**Affiliations:** 1 Department of Social Work, Rajagiri College of Social Sciences Autonomous, Kalamassery, IND; 2 Department of Library Science, Liquid Propulsion System Centre, Thiruvananthapuram, IND; 3 Department of Library Science, Marian College Kuttikkanam Autonomous, Kuttikkanam, IND; 4 Department of Psychology, Prajyoti Niketan College, Pudukad, IND; 5 Department of Social Work, Marian College Kuttikkanam Autonomous, Kuttikkanam, IND

**Keywords:** citespace, biblioshiny, bibliometric analysis, online addiction, social media addiction

## Abstract

Social media addiction is a behavioral dependency characterized by excessive and compulsive use of social media platforms, leading to negative impacts on various aspects of an individual's life. Bibliometric analysis is a research method used to quantitatively analyze academic literature, such as articles, books, and conference papers. It involves the application of statistical and mathematical tools to study the patterns and trends in scientific publications. This bibliometric study provides a comprehensive analysis of the literature on social media addiction, revealing patterns and dynamics within the field. Utilizing Web of Science for bibliographic data, the study employs advanced bibliometric tools like Biblioshiny and CiteSpace to map the scientific landscape. Annual scientific production, top contributing authors, key sources, trending topics, and thematic maps were identified using Biblioshiny. Additionally, network visualizations, such as co-citation networks of authors, time zone network visualizations of keyword co-occurrence, and timeline network visualizations of country collaborations, were created using CiteSpace. Our findings present an increasing trend in publications over the years, highlighting a growing recognition of social media addiction's significance. We detail the most relevant authors and sources, pinpointing key contributors and influential journals that shape the discourse. Trend topics analysis uncovers the prevalent themes, with "internet addiction" and "adolescents" at the forefront, reflecting the field's concentration on the younger population. The thematic map categorizes the research into motor themes (driving research areas), basic themes (fundamental and well-established areas), and niche themes (specialized and emerging topics), providing insight into the central and evolving topics. The study also delves into the co-occurrence of all keywords and the co-citation of authors, illustrating the interconnected nature of the research community. A timeline network visualization of country collaborations underscores the global scope of research efforts. Importantly, the study identifies critical research gaps such as underexplored demographics and emerging digital concerns and discusses practical implications, including the need for targeted intervention programs and informed policy-making. Collectively, this study charts the trajectory of social media addiction research and lays a foundation for future explorations to address identified lacunae.

## Introduction and background

In recent years, the proliferation of social media has fundamentally altered the landscape of communication and information sharing [1]. Platforms such as Facebook, Instagram, Twitter, and TikTok have woven themselves into the fabric of everyday life, influencing how individuals interact, form relationships, and consume content [2,3]. These communication tools have been significantly embraced, taking their place in the everyday lives of billions [4]. While these platforms have brought about numerous benefits, including enhanced connectivity and access to information, they have also introduced new challenges, particularly related to excessive use and addiction. This phenomenon of social media addiction has become a significant area of concern, prompting extensive academic research to understand its causes, consequences, and potential solutions.

Social media can easily become an unwelcome guest in our lives, constantly vying for our attention. While it offers connection and entertainment, excessive use can morph into addiction [5]. This is characterized by an uncontrollable urge to check social media, neglecting work or relationships in favor of scrolling. The constant need for approval and the dopamine hits from likes and comments fuel this dependence [6,7]. Social media addiction, also called problematic social media use or compulsive internet communication, is not just alarming but a contemporary dilemma of our times [8]. It is defined as specific diagnostic criteria or behaviors associated with addiction to social networking platforms leading to destructive consequences in one’s own life, including personal and professional life [9,10]. The primary reason for addiction is impossible to reject pleasure as a vital factor since every like, comment, or share contributes to an increase in dopamine production [11]. In contemporary society, a smartphone has become essential for full participation and interaction, seamlessly integrated into daily routines and providing vital access to connectivity [3].

The severity of social media addiction varies greatly. While someone may go hours on end simply scrolling through their feeds without concentrating on the content, someone else might be anxious and feel distraught when they cannot use their preferred platform [7,12]. However, it can be harmful to sleep schedules and cognitive and emotional work and damage real-life connections with others, leaving one feeling increasingly alienated and upset [13]. Despite most of the negative aspects of social media addiction, it is difficult for many people to break free of the consequences [14]. Fear of missing out (FOMO), social comparison, and validation need to fuel compulsive activities make it difficult to stop constantly checking the phone or computer.

Social media addiction has been a growing concern, particularly with the widespread adoption of platforms such as Facebook, Instagram, and Twitter. Studies have shown that social media addiction can lead to significant psychological and behavioral issues. For instance, Valakunde and Ravikumar developed a machine learning application to predict social media addiction, highlighting its prevalence among the youth [15]. LaRose et al. discussed the compulsive nature of social networking, comparing it to traditional media habits and examining the potential harmful consequences of such behaviors [16]. Furthermore, Sriwilai and Charoensukmongkol found that social media addiction negatively impacts mindfulness and coping strategies, leading to increased emotional exhaustion [17]. Andreassen et al. identified narcissism and low self-esteem as significant predictors of social media addiction, reflecting the need to feed one's ego and counteract negative self-evaluations [18]. These findings underscore the multifaceted nature of social media addiction, influenced by psychological traits, social behaviors, and the inherent design of social media platforms.

The landscape of academic research continuously changes, and the use of bibliometric analysis offers discovery power in relation to the trends, scope, and impact of scholarly literature in a particular domain of research [19,20]. In psychology and technology, the debate about addiction to social media has not only reached new heights in terms of its societal significance, but also, the seriousness with which the matter is taken has increased dramatically. The use of bibliometric analysis alongside innovative software packages, including Biblioshiny and CiteSpace, therefore provides a scholarly framework to examine the complex publication pattern that outlines the concern for social media addiction [21,22]. In detail, researchers using bibliometric techniques can identify patterns and commonalities and visualize the direction that research into this domain has taken over the decades.

A more user-friendly alternative to the R programming language is Biblioshiny which equips researchers with the necessary resources to conduct robust bibliometric analyses [23]. Using its simple interface, scholars can obtain pertinent bibliographic information from an array of academic databases, clean and preprocess datasets in a few clicks, and create visually appealing graphs that shed light on the academic landscape of social media addiction [24,25]. In tandem with Biblioshiny, CiteSpace, a dedicated bibliometric analysis instrument, goes a step further by allowing more sophisticated network analysis and visualization [26,27]. By constructing citation networks as well as networks of co-authorship and conceptual clusters, CiteSpace assists researchers in identifying patterns that might go unnoticed and crucial works to be cited and in tracing the dissemination of concepts in the social media addiction literature [28,29]. Through our bibliometric analysis, we seek to contribute to a deeper understanding of social media addiction and its multifaceted implications for individuals, society, and technology. By uncovering patterns and gaps in the existing literature, we hope to inform future research agendas, interventions, and policy initiatives aimed at addressing the complex challenges posed by social media addiction in the digital age.

This study aims to explore several pivotal research questions to deepen the understanding of social media addiction within the academic landscape. Firstly, we seek to identify the key thematic clusters that have emerged within scholarly literature on social media addiction and examine how these themes have evolved over time. Secondly, the study will highlight the most influential publications that have shaped the field, aiming to understand their impact and contributions. In addition, we will investigate the authors who have been most instrumental in advancing research in this area. Another focus of our inquiry will be to assess how research on social media addiction has spread across different geographic regions. Finally, we intend to pinpoint the key research gaps and propose areas for future exploration to guide subsequent studies in the field of social media addiction.

Biblioshiny and CiteSpace help answer research questions by identifying thematic clusters and tracking research theme evolution. Biblioshiny highlights annual scientific production, top authors, key sources, trending topics, and thematic maps. CiteSpace creates network visualizations, including co-citation networks of authors, keyword co-occurrence networks, and timeline visualizations of country collaborations. Together, these tools provide a comprehensive analysis of research trends and connections.

## Review

Materials and methods

We utilized the Web of Science core collection to access scientific papers relevant to our study [30]. Our search focused on the keywords "Social Media" and "Addiction," without any language restrictions. This approach aimed to capture a comprehensive and diverse set of studies from various linguistic backgrounds. The inclusion of papers in multiple languages ensured a broad perspective and minimized bias toward English-only publications. However, it also posed challenges in terms of accurate interpretation and analysis. To address this, we employed translation tools when necessary to ensure the accurate inclusion and understanding of non-English papers. We considered only journal articles, collecting a total of 1528 documents from 456 different sources. The search strategy involved using the Web of Science core collection. We combined the keywords "Social Media" and "Addiction" using the Boolean operator AND to ensure the search results included studies addressing both concepts. Additionally, we applied filters to limit the results to journal articles, spanning from 2010 to 2024. The search was not limited to the title, abstract, and keywords but included full texts to ensure a comprehensive collection of relevant documents. This approach helped capture studies that might have discussed social media and addiction in more detail within the full text. The outcomes were saved as a CSV file, and we used Biblioshiny and CiteSpace software to conduct a bibliometric analysis of the data.

Critical aspects of the investigation

Table 1 presents a comprehensive overview of research trends and characteristics within social media addiction research from 2010 to 2024. Over this period, a substantial body of literature consisting of 1528 documents from 456 sources has been examined, indicating a broad and diverse range of scholarly engagement with the topic. The annual growth rate of 41.18% was calculated by examining the increase in the number of published documents each year over the 14-year period. This rate reflects how much the body of literature has expanded annually, indicating a growing interest in the topic. The average document age of 2.92 years was determined by calculating the difference between the publication year of each document and the current year and then averaging these differences across all documents. This metric helps to illustrate the recency of the research, showing that the field is dynamic and evolving. The average of 14.91 citations per document refers to the average number of times each document has been cited, considering all documents in the dataset. This high citation average suggests that the documents in this field are heavily referenced, indicating significant scholarly engagement and impact. We also identified more than 37,368 references, 1511 Keywords Plus terms, and 2189 author keywords. Keywords plus terms are phrases that frequently appear in the titles of an article's references but not in the title of the article itself, while author keywords are the keywords provided by the authors of the articles. These were analyzed to understand the major themes and trends in the research. The significance of these keywords lies in their ability to highlight the core topics and emerging trends within the literature, offering insights into the focus areas and evolution of the research field. The recorded involvement of 5055 authors, with only 95 single-authored documents, indicates high research collaboration in this field. On average, there are 4.45 co-authors per document, and 23.56% of these documents involve international collaboration, highlighting the global interest and cooperative nature of this research area. The collection of 1528 peer-reviewed articles emphasizes a strict filtration process and consistent efforts to maintain high academic quality. Overall, the results show that the literature on social media addiction is extensive, dynamic, and gaining popularity.

**Table 1:** Main information of the investigation The annual growth rate percentage is a statistical measure used to evaluate the change in the number of publications over time in a specific field or topic.

Description	Results
Main information about data	
Timespan	2010:2024
Sources (journals, books, etc.)	456
Documents	1528
Annual growth rate %	41.18
Document average age	2.92
Average citations per doc	14.91
References	37368
Document contents	
Keywords Plus (ID)	1511
Author's Keywords (DE)	2189
Authors	
Authors	5055
Authors of single-authored docs	95
Authors collaboration	
Single-authored docs	104
Co-authors per doc	4.45
International co-authorships %	23.56
Document types	
Article	1462
Article; early access	66

Results and discussion

Annual Scientific Production

**Figure 1:**
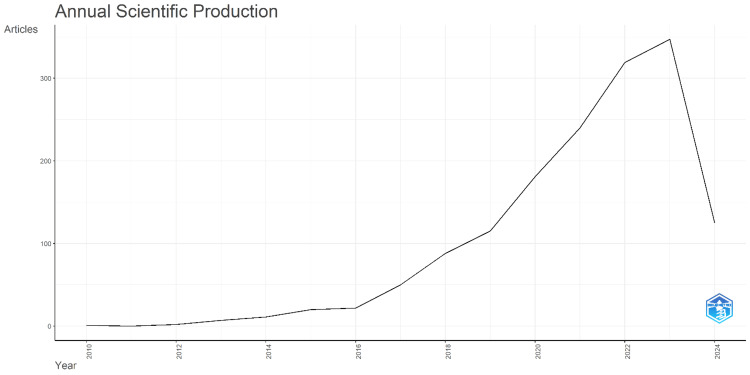
Annual scientific production

As shown in Figure 1, there is an increasing trend in the annual scientific production on addiction to social media. The increasing trend is a sign of increasing interest in research on this aspect, where there are more and more publications on the topic every year. The number of published articles has varied considerably from year to year: the increase was mainly between the three main years 2010, 2015, and 2023. In the years 2010 and 2011, scientific production was very scarce and amounted to 1 and 0 items respectively. After 2011, there was a continuous increase, which may have slowed in 2024 due to the data reception. In the following years, the production was still increasing, but the increases became less after the year 2023. Significantly more articles continued to be published each year until the peak year of 2023, 347 articles. The data for 2024 was incomplete, but it is likely that publications on the topic of addiction to social media will also continue to increase. This increasing trend shows that the recognition of the subject and the necessity to study it more are still developing.

Most Relevant Authors

Table 2 presents the authors who published the highest number of articles in social media addiction research. Mark D. Griffiths is the most relevant author, with 102 articles. Another author with the highest number of publications is Chung-Ying Lin with 41 articles, contributing to the understanding of social media addiction and its assessment, with a focus on health behaviors. Amir H. Pakpour, with 30 articles, touched the intersection of psychological health and digital behavior and its implications on well-being. Zsolt Demetrovics, with 23 articles, researched various addictive behaviors including the problematic use of the Internet and social media. Ofir Turel and Christian Montag, with 22 and 19 articles, respectively, highlighted the psychological and neurobiological sources of technology addiction or user’s personality traits and neurobiological features affecting digital media use. Within biological origins, Marc N. Potenza researched its aspects in addictions and their extension to technology use. I-Hua Chen examined digital media and mental health issues. Daria J. Kuss, with 14 articles published, explored the cognitive and behavioral spheres of online behaviors and related issues, while Kagan Kircaburun investigated risk factors for digital additions and their mechanisms. These authors have formed the current understanding of social media addition and opened many fields in social media addiction research, which allows if to cover the most relevant points of it.

**Table 2:** Most relevant authors

Authors	Articles
Griffiths MD	102
Lin CY	41
Pakpour AH	30
Demetrovics Z	23
Turel O	22
Montag C	19
Potenza MN	18
Chen IH	17
Kuss DJ	14
Kircaburun K	13

Most Relevant Sources

Table 3 presents a comprehensive picture of the most prolific sources that have served the dissemination of knowledge on social media addiction. Notably, Computers in Human Behavior with 88 articles shows the most significant contribution, which is not surprising given its status as the main source of the scholarly conversation. The International Journal of Environmental Research and Public Health is the next most renowned source for the research on social media addiction, totaling 83 articles. It is rather expected as a significant amount of research in the field is conducted from the perspective of public health. Another prominent source is the International Journal of Mental Health and Addiction, and the Current Psychology, with 54 and 52 articles, respectively. The other two multidisciplinary sources, Frontiers in Psychiatry and Frontiers in Psychology, have published 47 and 43 articles, respectively. The sources Addictive Behaviors, Journal of Behavioral Addictions, and Journal of Medical Internet Research contributed 40, 32, and 28 articles, respectively. PLOS ONE, with 27 articles, completes the list, highlighting the importance of the platform as a notable venue for open-access publishing. Overall, this extensive list of sources overall demonstrates the diversity of platforms, where research on social media addiction has been published over the last decade. This diversity and universality of the sources employed by researchers indicate the high level of interdisciplinary nature and the high level of collaboration in the field.

**Table 3:** Most relevant sources

Sources	Articles
Computers in Human Behavior	88
International Journal of Environmental Research and Public Health	83
International Journal of Mental Health and Addiction	54
Current Psychology	52
Frontiers in Psychiatry	47
Frontiers in Psychology	43
Addictive Behaviors	40
Journal of Behavioral Addictions	32
Journal of Medical Internet Research	28
PLOS One	27

Trend Topics

**Figure 2:**
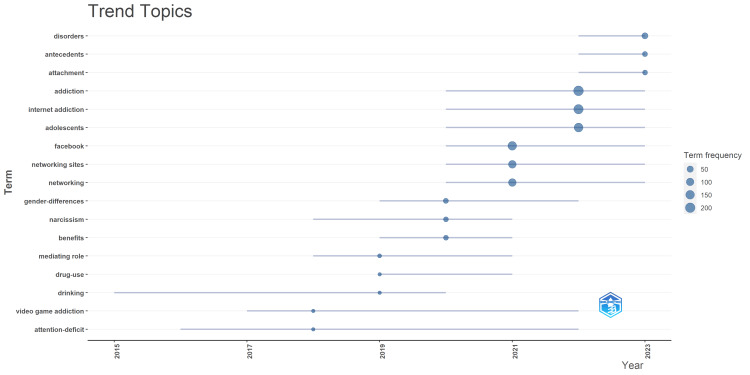
Trend topics

Figure 2 illustrates the frequency of specific terms found in research on social media addiction conducted between 2015 and 2023. One of the most interesting points stemming from Figure 2 includes an increase in a majority of terms and further suggests a growing concern surrounding the understanding and control of onset social media addiction. Figures such as “addiction,” “internet addiction,” “adolescents,” and “Facebook” account for the bulk, and more recently, a lump share of research, indicating a concern as researchers concentrate on the use of social media amongst younger users in particular. The continued rise in “disorders,” “antecedents,” and “attachment” illustrates a widening of the discipline and the concerned surrounding the presenting problems and the antecedents. In addition, a decrease in “video game addiction” could suggest as new or more pressing digital issues become prevalent, and the term is seen to dwindle in frequency. Others, with even less tender and even more erratic growth, include “gender differences” and “attention-deficit,” which speaks to specific areas. Based on data up until 2013, Figure 2 suggests specific trends and offers a current outlook on the complexity of the matter and suggests that the issue underlying levels within social media addiction interlink with documented trends drawn from the address pattern of drug addiction.

Thematic Map

**Figure 3:**
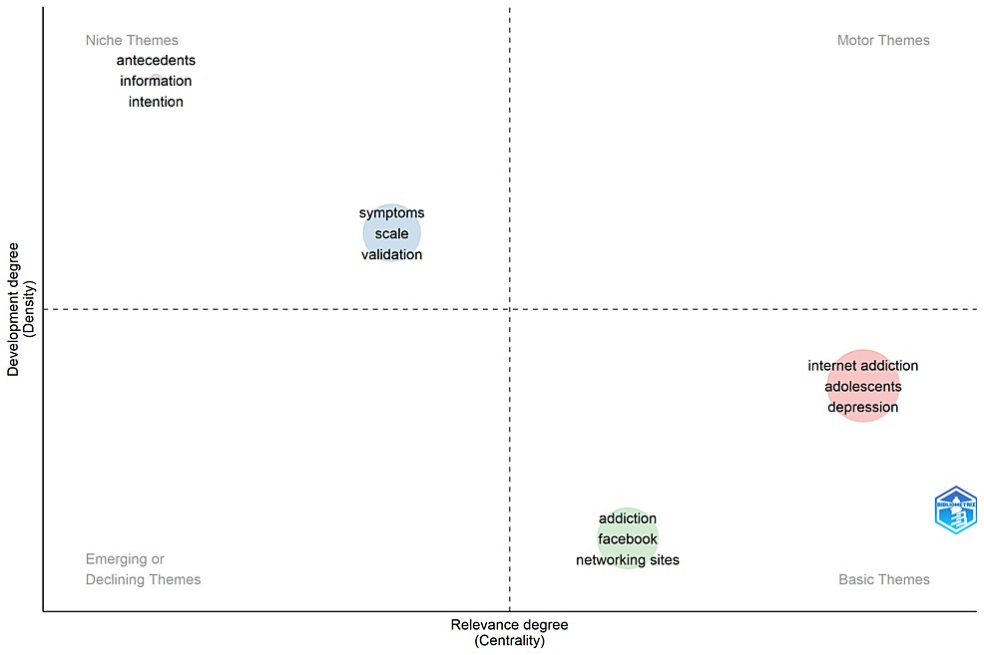
Thematic visualization of keywords

Figure 3 depicts a Porter strategic diagram that categorizes themes based on their centrality and development in the research body. The thematic map contains four quadrants, with centrality, referred to as relevancy represented on the horizontal axis and development, referred to as density, represented along the vertical axis. The positions of terms in the quadrants help to assess the importance of the term and the evolution o each theme in the research field. Motor Themes are highly central themes that are well developed and crucial to the research domain. Basic Themes: The bottom-right quadrant contains the terms “internet addiction,” “adolescents,” “depression”, “addiction,” “Facebook,” and “networking sites”. These themes are highly central but less dense than those in the Motor Themes quadrant. This implies that the themes are central to human addiction and Facebook but have been less heavily researched. Niche Themes: The top-left quadrant contains “antecedents,” “information,” and “intention.” These themes are well-developed but less centrally situated. This means that even though they are not at the center of the research topic, they have been widely investigated. They probably represent specialized research areas that are less important to the entire research community but significant to certain audiences. Emerging or Decreasing Themes: The bottom-left quadrant is invisible. Generally, such a quadrant would be used to describe non-centrally and non-densely situated themes. These could be new topics that are only starting to attract attention or related to past themes that are no longer important. In general, the lack of terms in this quadrant suggests that there were no themes that had low centrality and low development across the present research.

Network Visualization of Co-citation of Authors

**Figure 4:**
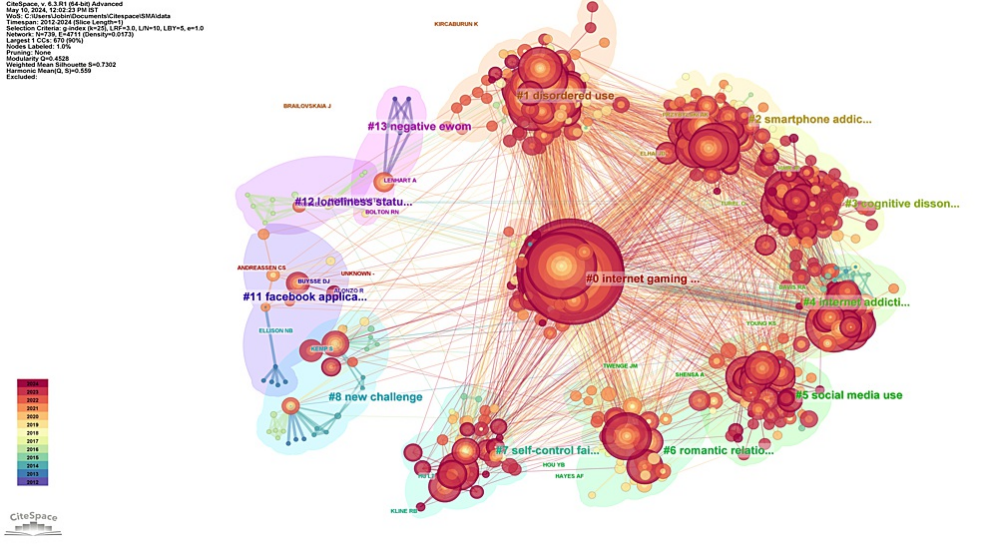
Network visualization of co-citation of authors

Figure 4 presents the clustered network visualization of the co-citation of authors. In this regard, 12 clusters with a centric theme can hint at the main areas of focus. Each cluster in the presented figures highlights particular themes present in addiction research and outlines the key authors and the chief works that influenced the field. The silhouette value, which measures the cohesion and separation of the clusters, plays a significant role in understanding the robustness of these clusters. A high silhouette value (close to 1) indicates well-defined and distinct clusters, while a lower value indicates less distinct grouping. Cluster 0 - Internet Gaming Disorder: With the most extensive number of members and 113, it features internet gaming disorder and problematic social media use. This cluster presents a high silhouette value of 0.746, denoting cohesion within the cluster. Leading contributors suggest that the cluster is well-researched, with that work highlighted by leading figures such as Andreassen CS and Kuss DJ, who present research on how playing a game is linked with addictive behavior on the internet. Cluster 1 - Disordered Use: Also with a number of 113 members, this cluster features disordered use of social media and includes a silhouette of 0.585. The major contributors include Kircaburun K and Brailovskaia J, who suggest that the psychological problems and addictive patterns related to social media are the main focus of the cluster. Cluster 2- Smartphone Addiction: With 97 members, this cluster has a silhouette of 0.691, presenting smartphone addiction. The leading authors like Elhai JD and Przybylski AK argue that smartphone addiction is various, and the overuse of mobile devices increases the likelihood of individuals facilitating addictive behaviors. Cluster 3 - Cognitive Dissonance: The number of 87 members explores cognitive dissonance with a silhouette of 0.711, and the main cited authors are Turel O and Hair JF. The cluster suggests that internet users, even those knowledgeable of its consequences, are still engaging. Cluster 4 - Internet Addiction features 64 members and a silhouette of 0.748, presenting the internet addiction aspects. The main contributors, Young KS and Davis RA, argue that the Internet is a compulsive tool and the addiction treatment center is equipped to help. Cluster 5 - Social Media Use: With 54 members, this cluster has 54 members that explore social media use, and its correlation development has a silhouette of 0.751. Shensa A and Twenge JM deny the strong link to social media use or other issues with Facebook and Instagram. Cluster 6 - Romantic Relationship: 50 members explore how social media has influenced romantic relationships with a silhouette of 0.843. Hayes AF and Hou YB Creator note that social media has become a part of everyday life and has taken the place of possible romantic rendezvous. Cluster 7 - Self-Control Failure: 36 members explore self-control failure with a silhouette of 0.813. Is it possible to control your time online, they ask, or is your inaction the primary cause of addiction? Cluster 8 - New Challenge: 26 members, refer to the new challenge presented with new social media tools, and the silhouette of 0.938. Ellison NB and Kemp S Present the results of the global survey on digital trends in outlets becoming more popular around the world. Cluster 11 - Facebook Application is one clustered with a notion that the authors place the hype with Facebook, and its addictiveness with 12 members that has a silhouette of 0.953. Buysse DJ and Alonzo R present the recent survey of American teenagers who were asked, among other things, about their Facebook usage. Cluster 12 - Loneliness Status denotes the linkage with the total number of 11 members and the silhouette of 0.967. Morahan-Martin J and Russell D have presented the design and characteristics of the format and explain why people use it to join various discussions on important topics. Cluster 13 - Negative EWOM: Final research cluster is the clustered negative electronic word-of-mouth extension in seven members and the silhouette of 0.983 Lenhart A and O’Keeffe G help readers understand and avoid negative content and addiction.

Time Zone Network Visualization of Co-occurrence of All Keywords

**Figure 5:**
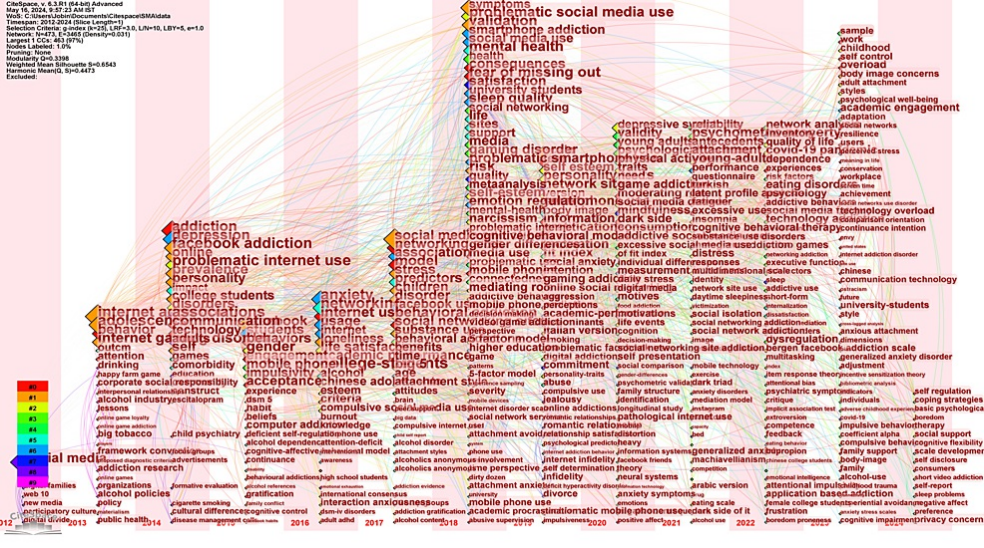
Network visualization of co-occurrence of all keywords

Figure 5 depicts the time zone network visualization showing the dynamic landscape of social media addiction research over time, with keywords co-occurring in various research areas. The network consists of 10 clusters and each cluster represents a concentration of research themes and their evolution. Cluster #0 - Social Media Fatigue: Dominating the research field with 89 members, this cluster signifies a comprehensive exploration of social media fatigue. Keywords such as 'addiction', 'networking sites', and 'internet use' are frequent, suggesting that studies within this cluster examine the effects of excessive social media use on individuals' well-being and its association with addiction-like symptoms. Cluster #1 - Psychometric Evaluation: With 67 members, this cluster is essential for developing reliable and valid measures for internet and social media addiction, especially among younger populations like adolescents. 'Internet addiction' and 'social media addiction' are prominent keywords, indicating efforts to quantify and diagnose these modern phenomena rigorously. Cluster #2 - Dark Triad: This cluster, including 65 members, looks at the darker aspects of personality (Dark Triad: narcissism, Machiavellianism, psychopathy) and how they relate to problematic social media use. 'Problematic social media use', 'problematic internet use', and 'depressive symptoms' are key terms, suggesting that this cluster is concerned with the psychological factors contributing to or exacerbating social media misuse. Cluster #3 - Eating Disorder Symptom: Encompassing 55 members, this cluster correlates social media addiction with eating disorders. The co-occurrence of 'health', 'social networking', and 'gaming disorder' within this cluster suggests a multidisciplinary approach, potentially looking at body image issues propagated by social media and their impact on health behaviors. Cluster #4 - Addictive Social Media Use: This cluster contains 53 members and zeroes in on the nature of addictive social media use. 'Validity', 'young adults', and 'reliability' are significant here, possibly indicating an interest in the development of robust assessment tools to identify and study addictive behaviors among young adult populations. Cluster #5 - Psychopathological Risk: With 48 members, the focus in this cluster is on the broader mental health risks associated with social media use. 'Networking', 'mental health', and 'impact' are important keywords, pointing toward studies that assess the overall mental health implications of sustained social media use. Cluster #6 - Academic Performance: Containing 42 members, this cluster investigates the effects of social media on academic performance. Frequently occurring terms like 'depression', 'anxiety', and 'smartphone addiction' imply a research nexus that looks at how social media use may contribute to stress and impact students' academic outcomes. Cluster #7 - Committed Relationship: With 18 members, this cluster explores how social media can affect committed relationships. 'Satisfaction', 'associations', and 'network sites' are prominent, indicating that research here might delve into how online interactions influence relational dynamics and satisfaction levels. Cluster #8 - Academic Performance: Although similar in theme to Cluster #6, this smaller cluster with 15 members also deals with academic performance but seems to take a slightly different angle, perhaps focusing more specifically on social media use and its cognitive and emotional implications in learning environments. Cluster #9 - Alcohol Industry: The smallest cluster, with 11 members, uniquely examines the intersection of the alcohol industry with social media. The keywords 'drinking', 'public health', and 'policy' suggest an analysis of how the alcohol industry uses social media and the consequent public health and policy implications. The visualization illustrates the intersection and divergence of various research themes over time, reflecting the multidimensional impact of social media on different facets of life. Each cluster's size and keyword density offer insight into the depth and focus of research conducted within each domain.

Timeline Network Visualization of Countries’ Collaborations

**Figure 6:**
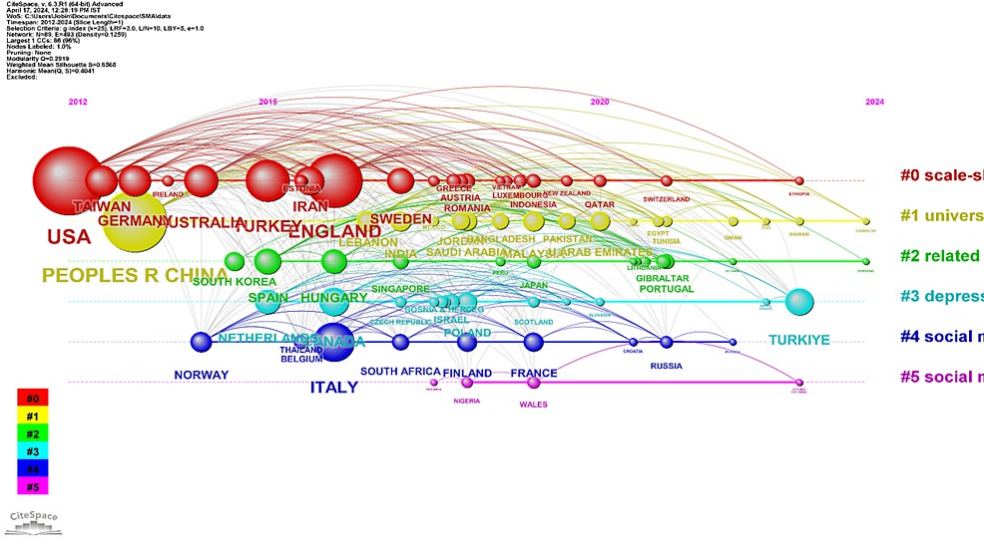
Timeline network visualization of countries’ collaborations

Figure 6 presents a timeline network visualization that maps collaborative efforts of various countries in researching social media addiction. The visualization shows six distinct clusters. Each cluster focuses on different aspects of social media-related studies and their international collaboration patterns. Cluster #0 - Scale-Short Form: As the largest cluster with 23 members, this group appears to concentrate on the development and application of abbreviated scales for measuring phenomena related to social media use, perhaps in special populations like patients with cancer. The United States, England, and Turkey are the most cited countries in this cluster, indicating they are key players in this research area. Cluster #1 - University Student: With 19 members, this cluster is highly cohesive (as indicated by the silhouette value) and focuses on university students and social media addiction. It might be examining the psychological impacts of technology on this demographic. The People's Republic of China, India, and Malaysia are the leading contributors, which suggests a significant research output on social media addiction among students in these countries. Cluster #2 - Related Risk: This cluster includes 15 members and studies the risks associated with social media, with particular reference to the online survey method as a research tool. Spain, Hungary, and South Korea are the top contributing countries, highlighting their involvement in international research on the risks of social media use, potentially intensified during events like COVID-19 self-isolation. Cluster #3 - Depression Anxiety: Comprising 14 members, this cluster's research seems to be associated with depression and anxiety as it relates to social media use. The most cited countries in this cluster are Canada, Turkiye (Turkey), and the Netherlands, which may be at the forefront of investigating the mental health consequences of social media usage. Cluster #4 - Social Media Fatigue: With 10 members, the focus here is on social media fatigue. Italy, Norway, and Finland are the leading collaborators in this cluster. The research likely revolves around the consequences of over-engagement with social media platforms like Instagram and Snapchat and the psychological phenomenon of FOMO. Cluster #5 - Social Media Sites Users Choice: The smallest cluster, with five members, looks into users' choices between different social media sites and how they balance utilitarian and informational needs. Wales, Nigeria, and Colombia are the most cited in this cluster, indicating that they may be exploring the decision-making processes behind social media use and the potential for addiction. Each cluster provides insights into the geographical distribution and collaborative efforts of countries investigating various aspects of social media use and its psychological impacts. The silhouette values indicate the internal consistency of the clusters, with higher values denoting more tightly knit research within the cluster. The most cited countries within each cluster likely reflect where significant research contributions are originating or where collaborative efforts are particularly strong.

Research gaps and practical implications

After analyzing trend topics and mapping themes in social media addiction research, several research gaps and practical implications were identified. The focus on adolescents in social media addiction research overshadows the need for studies on other demographics, particularly regarding gender differences and cultural impacts, suggesting a gap in the literature that could be filled with more targeted research. Additionally, the decline in research on "video game addiction" and limited exploration beyond platforms like Facebook points to a need for updated studies encompassing newer social media platforms and digital technologies. Specific psychological conditions such as "attention-deficit" are not as prominently featured, indicating room for more in-depth studies on how various neurodevelopmental conditions intersect with social media usage. Despite continued interest in "narcissism" and "benefits," research into the positive aspects of social media use-its role in community, social support, and education-appears to be insufficient. The trends also suggest that longitudinal studies examining the antecedents and development of social media addiction over time are crucial for understanding its long-term effects.

From a practical standpoint, these findings imply a significant need for the development of intervention and prevention programs, especially for young users who are at increased risk of addiction. Policies and regulations could be informed by the basic themes of "Facebook" and "networking sites," addressing the addictive potential of these platforms. Education campaigns based on the motor theme of "internet addiction" could raise public awareness about addiction signs and healthy internet habits. Clinicians could benefit from the research on "antecedents" and "information," using it to craft better screening tools and interventions. In technology development, there is an opportunity for platforms to integrate research findings to design features that prevent addiction or help manage use. 

Specifically, policy-making could be informed by the identified gaps, leading to regulations that limit excessive use and encourage healthier online behaviors. Educational programs could incorporate findings on the impact of social media on mental health, providing students with strategies to balance their online and offline lives. Clinical interventions could be tailored based on the psychological conditions linked to social media addiction, offering more personalized treatment plans. Finally, the absence of emerging or declining themes indicates that research funding and direction could pivot towards exploratory studies in uncharted territories of social media addiction, broadening the field's understanding and informing practical solutions to address this growing concern.

Limitations of the study

Firstly, the reliance on the Web of Science core collection may have excluded relevant studies indexed in other databases, potentially narrowing the scope of our findings. Additionally, while our quantitative analysis provides valuable insights into research trends, it lacks the depth of qualitative analysis that could further illuminate the nuanced perspectives and subjective experiences related to social media addiction. This limitation suggests that future studies should incorporate qualitative approaches to provide a more comprehensive understanding of the topic.

## Conclusions

This bibliometric analysis reveals a rapidly expanding field of research on social media addiction, with a notable focus on its impact on the younger generation. This is evident from the prevalence of studies concentrating on adolescents and young adults, reflecting concerns about the susceptibility of these age groups to addictive behaviors related to social media use. The current research networks illustrate a number of highly productive authors and primary publications that drive the discussion and research agenda. However, there are still many unanswered questions, as the existing literature does not cover all areas under examination. Addressing knowledge gaps, such as the effects of social media addiction on different demographics, gender differences, and cultural impacts, could provide a more comprehensive understanding of the issue. Exploring the intersection of social media use with specific psychological conditions, such as attention-deficit disorders, and studying newer social media platforms and digital technologies could also yield valuable insights. Addressing these gaps could have significant practical and theoretical impacts. Practically, it could lead to the development of more targeted intervention and prevention programs, informed policy-making to regulate excessive use, and enhanced educational programs to promote healthier online behaviors. Theoretically, it would enrich the existing body of knowledge, offering a more nuanced view of social media addiction's multifaceted nature. Global analysis of cooperation patterns proves the universal nature of this topic across numerous geographies and countries. The results demonstrate a critical need for interventions, policy formation, and educational program development to address social media addiction issues comprehensively. In conclusion, this study highlights the urgent need for new research to enhance our understanding and prompt timely actions against the growing challenge of social media addiction.
